# Patency of Individual and Sequential Coronary Artery Bypass in Patients with Ischemic Heart Disease: A Meta-analysis

**DOI:** 10.21470/1678-9741-2018-0284

**Published:** 2019

**Authors:** Zeshu Li, Luqi Liu

**Affiliations:** 1 Department of Thoracic and Cardiovascular Surgery, Shandong Provincial PKUcare Luzhong Hospital, Zibo, Shandong, People's Republic of China.; 2 Department of Cardiac Surgery, Shandong Provincial Qianfoshan Hospital, Shandong University, Jinan, Shandong, People's Republic of China.

**Keywords:** Coronary Artery Bypass, Coronary Artery Disease, Myocardial Ischemia, Hospital Mortality, Meta-Analysis as Topic

## Abstract

**Objective:**

To evaluate the patency of individual and sequential coronary artery bypass in patients with ischemic heart disease.

**Methods:**

We searched PubMed, Cochrane Library, Excerpta Medica Database, and ClinicalTrials.gov databases for controlled trials. Endpoints included graft patency, anastomosis patency, occluded rates in left anterior descending (LAD) system and right coronary artery (RCA) system, in-hospital mortality, and follow-up mortality. Pooled risk ratios (RRs) and standardized mean difference (SMD) were used to assess the relative data.

**Results:**

Nine cohorts, including 7100 patients and 1440 grafts under individual or sequential coronary artery bypass. There were no significant differences between individual and sequential coronary artery bypass in the graft patency (RR=0.96; 95% CI=0.91-1.02; *P*=0.16; *I*^2^=87%), anastomosis patency (RR=0.95; 95% CI=0.91-1.00; *P*=0.05; *I*^2^=70%), occluded rate in LAD system (RR=1.03; 95% CI=0.92-1.16; *P*=0.58; *I*^2^=37%), occluded rate in RCA system (RR=1.36; 95% CI=0.72-2.57; *P*=0.35; *I*^2^=95%), in-hospital mortality (RR=1.57; 95% CI=0.92-2.69; *P*=0.10; *I*^2^=0%), and follow-up mortality (RR=0.96; 95% CI=0.36-2.53; *P*=0.93; *I*^2^=0%).

**Conclusion:**

No significant differences on clinical data were observed regarding anastomosis patency, occluded rate in LAD system, occluded rate in RCA system, in-hospital mortality, and follow-up mortality, indicating that the patency of individual and the patency of sequential coronary artery bypass are similar to each other.

**Table:** 

Abbreviations, acronyms & symbols				
CABG	= Coronary artery bypass grafting		PDA	= Posterior descending artery
CIs	= Confidence intervals		PRISMA	= Preferred Reporting Items for Systematic Review and Meta-analysis
Cl	= Control		RCA	= Right coronary artery
CT	= Computed tomography		RRs	= Risk ratios
EMBASE	= Excerpta Medica database		SMD	= Standardized mean difference
LAD	= Left anterior descending			
NOS	= Newcastle Ottawa Scale			

## INTRODUCTION

Ischemic heart disease is currently the leading cause of death worldwide and will account for 14.2% of all deaths by 2030. Also, it is a major contributor to societal costs of cardiac disease^[1]^. Coronary artery bypass grafting (CABG) is one of the common surgeries for cardiac patients, which is the best treatment for advanced ischemic heart disease^[2]^. The sequential grafting technique in CABG was introduced by Flemma et al.^[3]^ in the 1970s. Since then, different methods of anastomosis such as individual or sequential grafts have been used. However, the efficacy of these methods is controversial.

Our meta-analysis was undertaken to analyze the efficacy of individual and sequential grafts used in patients with ischemic heart disease and under CABG.

## METHODS

Using the keywords "coronary artery bypass grafting", "individual graft", "sequential graft", and "ischemic heart disease", we searched PubMed, Cochrane Library, Excerpta Medica database (EMBASE), and ClinicalTrials.gov databases and got data from inception to February 25, 2018. The search was restricted to studies with humans and had no restrictions in language. In addition, references from randomized trials and relevant reviews that were not identified in the database search were hand-searched.

The following inclusion criteria were applied: (1) patients with ischemic heart disease and under CABG; (2) cohort trials that compared the efficacy of individual and sequential coronary artery bypass; and (3) clinical outcomes reported, such as patency rate, blood flow, and the incidence of death. Reviews, meta-analyses, and observational studies were excluded. The meta-analysis was conducted according to the Preferred Reporting Items for Systematic Review and Meta-analysis (PRISMA) guidelines^[4]^.

Two investigators independently extracted data from the relevant sources. Authors were contacted when data were incomplete or unclear and conflicts were resolved by discussion. Baseline demographic and quality characteristics (sample size, age, sex, community, and follow-up) of patients were collected from the eligible studies. The patency rate of graft and anastomosis, blood flow, and mortality were recorded. Newcastle Ottawa Scale (NOS) was used to assess the quality of included literatures based on recommendations from a non-randomized methodological level^[5]^.

### Statistical Analysis

Binary classification variables of the clinical endpoints were measured using the risk ratios (RRs) with 95% confidence intervals (CIs). The continuous variables of the clinical endpoints were determined by standardized mean difference (SMD) with 95% CIs. Two-sided *P*-values<0.05 were considered statistically significant. Heterogeneity was assessed by the Cochran Q test and *I*^2^ statistic, and Cochran's *P*<0.10 and *I*^2^>50 were considered indicative of significant heterogeneity. Pooled analyses were conducted using a fixed effect model, whereas a random effect model was used if there was significant heterogeneity. Publication biases were assessed by funnel plot analysis and Egger's test. Data analysis was conducted using the RevMan 5.3 software (Nordic Cochrane Centre, Cochrane Collaboration, 2013), and sensitivity analysis was performed by the Stata 11.0 software (StataCorp, College Station, Texas, USA).

## RESULTS

### Data Search Results

**Fig. 1:**
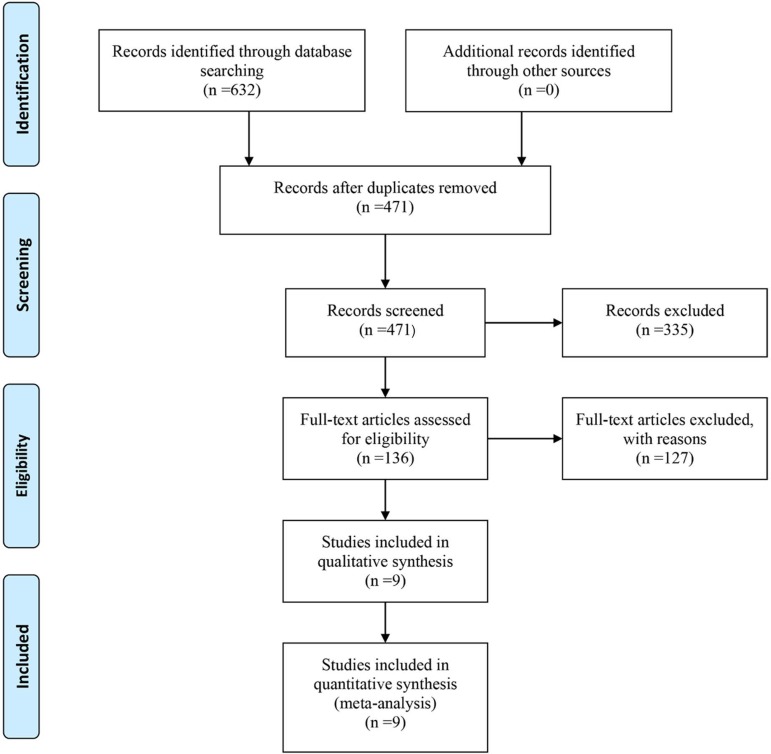
Flowchart showing the progress of data selection.

We identified nine trials^[6-14]^ out of 632 records that satisfied our inclusion criteria, as shown in the selection procedure depicted in Figure 1. A total of 7100 patients and 3060 grafts under individual coronary artery bypass and 7380 grafts under sequential coronary artery bypass were included. Baseline characteristics and quality assessment according to NOS is presented in Table 1. All clinical trials included in our study were middle to high quality cohort studies, with seven to nine NOS scores.

**Table 1:** Baseline characteristics and quality assessment.

References	Community	Comparability			Assessment method	Follow-up time	Quality assessment (NOS)				
		**Age**	**Male (%)**	**Other factors**			**Rate (%)**	**Selection**	**Comparability**	**Outcome**	**Total**
Takazawa et al.^[13]^, 2015	Saitama International Medical Center in Japan	71±8	63.4	_	Angiography	14.7±17.5	_	4	1	2	7
Fukui et al.^[8]^, 2012	Sakakibara Heart Institute, Tokyo, Japan	67.2±10.4/67.0±10.7	76.5/85.2	Body surface area	Angiography	12.1(221)	50.9	4	2	1	7
Samano et al.^[12]^, 2017	Orebro University Hospital, rebro, Sweden.	75.6±8.5	74.6/81	Body mass index, hypertension	Angiography	72	93.8	4	2	3	9
Ji et al.^[10]^, 2017	Zhongshan Hospital Fudan University, China	63.6±8.5/62.9±9.4	87.5/90	Smoking history, diabetes mellitus	Computed tomographic angiography	27.0±7.3	_	4	2	2	9
Gao et al.^[9]^, 2010	Patients operated on by a single surgeon	63.6±10.3	89	_	Angiography	26.4±23.6	_	4	2	1	7
Kim et al.^[11]^, 2011	Asan Medical Center	63.7±8.3/62.9±8.3	69.6/69	Hypertension, diabetes mellitus	Dual-source CT	14.8	_	4	2	2	8
Farsak et al.^[7]^, 2003	_	55.2±9.3	87	Atherosclerotic risk factors	Angiography	55.4±17.6	_	3	2	2	7
Vural et al.^[14]^, 2001	Yuksek Ihtisas Hospital in Turkey	49±8	89	Atherosclerotic risk factors	Angiography	69.6	_	4	2	2	8
Christenson et al.^[6]^, 1998	_	58.2±9.2	81	Hypertension, hyperlipidemia, diabetes	Angiography	76	99.10%	3	2	3	8

CT=computed tomography; NOS=Newcastle Ottawa Scale

### Graft Patency

**Fig. 2:**
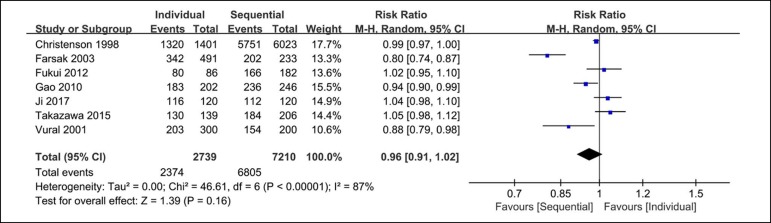
Forest plot of graft patency. CI=confidence interval

Seven clinical studies reported the results of graft patency. The analysis of the graft patency rate includes 2374 out of 2739 grafts from the individual coronary artery bypass group and 6803 out of 7210 from the sequential coronary artery bypass group. There are no significant differences between these groups (RR=0.96; 95% CI=0.91-1.02; *P*=0.16; *I* ^2^=87%) on the patency of grafts, as shown in Figure 2.

### Anastomosis Patency

**Fig. 3:**
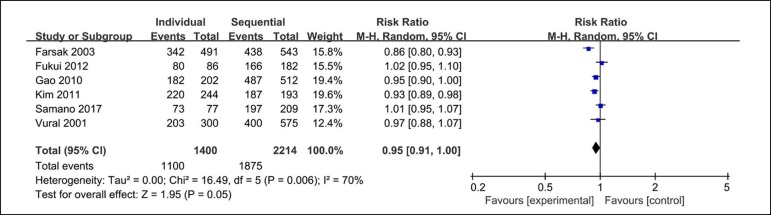
Forest plot of anastomosis patency. CI=confidence interval

There were six clinical studies showing the results of anastomosis patency. The analysis of anastomosis patency includes 1100 out of 1400 anastomosis from the individual coronary artery bypass group and 1875 out of 2214 from the sequential coronary artery bypass group. There are no significant differences between these groups (RR=0.95; 95% CI=0.91-1.00; *P*=0.05; *I* ^2^=70%) in the patency of anastomosis (Figure 3).

### Occluded Rate in Left Anterior Descending (LAD) System

**Fig. 4:**
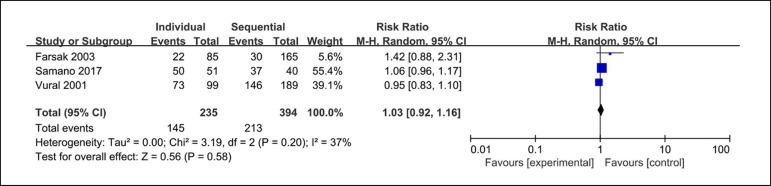
Forest plot of occluded rate in LAD system. CI=confidence interval; LAD=left anterior descending

Three clinical studies reported the occluded rate in LAD system. The analysis found the occluded rate in LAD system in 145 out of 235 LAD system anastomoses from the individual coronary artery bypass group and 213 out of 349 from the sequential coronary artery bypass group. There are no significant differences between these groups (RR=1.03; 95% CI=0.92-1.16; P=0.58; *I* ^2^=37%) in the occluded rate in LAD system, as demonstrated in Figure 4.

### Occluded Rate in Right Coronary Artery (RCA) System

**Fig. 5:**
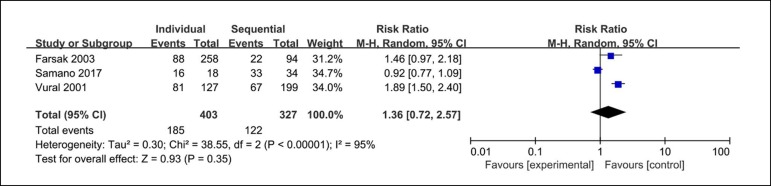
Forest plot of occluded rate in RCA system. CI=confidence interval; RCA=right coronary artery

The occluded rate in RCA system was reported in three clinical studies. The analysis found the occluded rate in RCA system in 185 out of 403 RCA system anastomoses from the individual coronary artery bypass group and 122 out of 327 from the sequential coronary artery bypass group. There are no significant differences between these groups (RR=1.36; 95% CI=0.72-2.57; P=0.35; I^2^=95%) in the occluded rate in RCA system, as shown in Figure 5.

### In-hospital Mortality

**Fig. 6:**
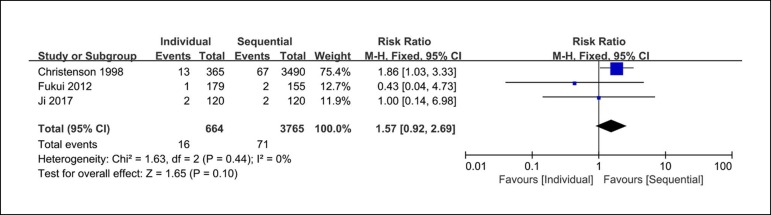
Forest plot of in-hospital mortality. CI=confidence interval

There were three clinical studies reporting the in-hospital mortality rates. The analysis of n of hospital mortality shows 16 of 664 patients from the individual coronary artery bypass group and 71 of 3765 from the sequential coronary artery bypass group. However, there are no significant differences between these groups (RR=1.57; 95% CI=0.92-2.69; *P*=0.10; *I* ^2^=0%) in the in-hospital mortality rates (Figure 6).

### Follow-up Mortality

**Fig. 7:**
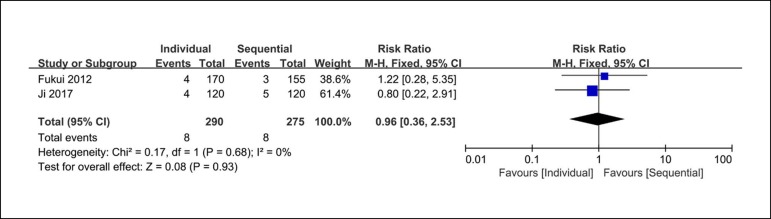
Forest plot of follow-up mortality. CI=confidence interval

There were two clinical studies reporting the follow-up mortality rates. The analysis of n of hospital mortality shows eight out of 290 patients from the individual coronary artery bypass group and eight out of 275 patients from the sequential coronary artery bypass group. There are no significant differences between these groups (RR=0.96; 95% CI=0.36-2.53; *P*=0.93; *I* ^2^=0%) in the follow-up mortality rates (Figure 7).

### Sensitivity and Publication Bias Analyses

**Fig. 8:**
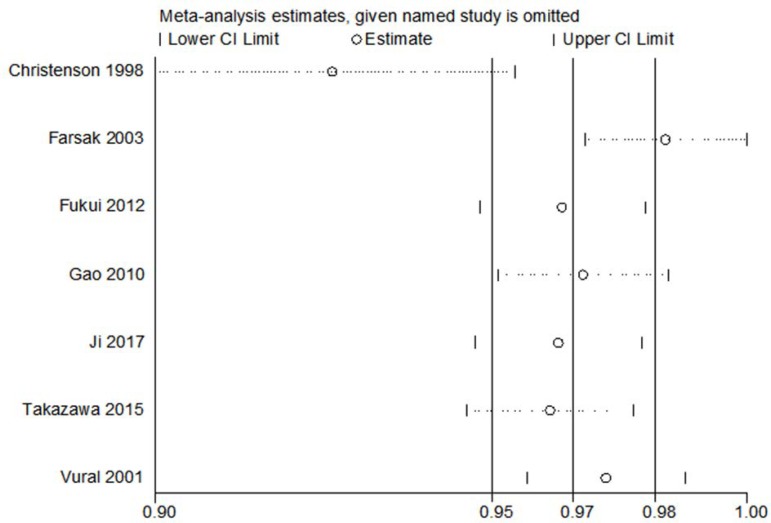
Sensitivity analysis of excluding each individual study. Cl=control

Sensitivity analysis was conducted by excluding each individual study. It was found that the study of Christenson^[6]^, in 1998, resulted in a significantly different result, as shown in Figure 8. A similar meta-analysis outcome was obtained, which demonstrated that our conclusion is stable, and this heterogeneity is not affected by the combined results. No significant evidence of publication bias was obtained using the Begg's test in the study endpoints, as shown in Table 2.

**Table 2:** Publication bias of the Begg's test.

Endpoints	*P*-value
Graft patency	1.00
Anastomosis patency	0.133
Occluded rate in LAD system	0.296
Occluded rate in RCA system	0.296
In-hospital mortality	0.296

LAD=left anterior descending; RCA=right coronary artery

## DISCUSSION

CABG has become the gold standard for the treatment of coronary artery disease involving multiple vessels, and it consists of on-pump CABG and off-pump CABG. In the early 1980s, two surgeons published their extensive series of off-pump CABG in patients who received grafts in the LAD and the main RCA, but with more limited and difficult grafting of coronary arteries on the posterior and lateral walls^[15,16]^. On-pump CABG provides a motionless operative field, but it can be associated with a number of complications, such as myocardial ischemic injury, strokes, coagulation, and inflammatory responses^[17,18]^. To the present, it has been reported that the advantage of sequential coronary artery bypass technology is that it can save grafts, reduce proximal anastomosis, shorten the operation time, provide a more complete vascularization, and have a satisfactory long-term patency rate^[19,20]^. It is more accurate to determine the direction and length of the bridge between anastomoses. There is a study showing that the proximal obstruction of the sequential bridge leads to the reduction of blood flow in multiple coronary arteries, resulting in a large area of myocardial infarction, and endangers the patient's life^[21]^. It is also considered that the distal end of the proximal end of the sequential bridge plays an important role in collateral circulation, and patients rarely have myocardial infarction^[22,23]^. In addition, some professors and their teams have found out that a sequential bridge can reduce the blood flow resistance of bridges, which reduces the mismatch of vascular resistance and increases the long-term patency rate.

The coronary circulation can be divided into left-dominant, right-dominant, and co-dominant systems. In a left-dominant system, the posterior descending artery (PDA) is supplied by the circumflex artery. In a right-dominant system, the PDA is supplied by the RCA^[24]^. In this meta-analysis, we included nine trials with a total of 7100 patients and 1440 grafts under individual or sequential coronary artery bypass. We found out that the individual and sequential coronary artery bypass associated show no significant differences in the graft patency, anastomosis patency, occluded rate in LAD system, occluded rate in RCA system, in-hospital mortality, and follow-up mortality. In a previous study, the patency of sequential coronary artery bypass was lower than of individual coronary artery bypass. In a meta-analysis of RCTs on almost 16,900 patients, it was found no difference in 30-day mortality^[25]^. Kowalewski et al.^[26]^ found out that 19,000 patients demonstrated no significant difference in short-term mortality. Another investigation in a recent meta-analysis of RCTs indicated no difference in patients with over a six-months follow-up (RR, 1.02; 95% CI: 0.86-1.22; *P*=0.81)^[27,28]^. In the present study, we included the newest clinical trials and compared the individual and sequential coronary artery bypass groups regarding grafts and anastomosis. Also, we performed sensitivity and publication bias analyses, which demonstrates that our analysis is stable and has no publish bias.

### Limitation

Nevertheless, there are some limitations in this meta-analysis. Firstly, the graft used in coronary artery bypass is not unified. Saphenous vein grafts and internal thoracic artery are both included in this study, which might have affected the reliability of the results. Secondly, several of the included clinical trials are cohort studies, instead of randomized clinical trials, which reduces the level of evidence. In addition, the generally different designs and characteristics of each trial might have also caused heterogeneity. Therefore, more rigorous, large-sample, international trials are needed to further confirm the results.

## CONCLUSION

In conclusion, no significant difference on clinical data were observed regarding anastomosis patency, occluded rate in LAD system, occluded rate in RCA system, in-hospital mortality, and follow-up mortality. The patency of individual and the patency of sequential coronary artery bypass are similar to each other.

**Table:** 

Authors' roles & responsibilities	
ZL	Substantial contributions to the conception or design of the work; or the acquisition, analysis, or interpretation of data for the work; final approval of the version to be published
LL	Substantial contributions to the conception or design of the work; or the acquisition, analysis, or interpretation of data for the work; final approval of the version to be published
