# ERRATUM

**DOI:** 10.21470/1678-9741-2018-0284e

**Published:** 2019

In the original article "Patency of Individual and Sequential Coronary Artery Bypass in Patients with Ischemic Heart Disease: A Meta-analysis", published in the Brazilian Journal of Cardiovascular Surgery 34.4, pages 420 to 427, in the article that the first unit of Zeshu Li belongs to Department of Thoracic and Cardiovascular Surgery, Shandong Provincial PKUcare Luzhong Hospital, Zibo, Shandong, People’s Republic of China and the second unit is Department of Cardiac Surgery, Shandong Provincial Qianfoshan Hospital, Shandong University, Jinan, Shandong, People’s Republic of China. It is the correct that the author of Zeshu Li first belongs to Department of Cardiac Surgery, Shandong Provincial Qianfoshan Hospital, Shandong University, Jinan, Shandong, People’s Republic of China and the second unit it's the Department of Thoracic and Cardiovascular Surgery, Shandong Provincial PKUcare Luzhong Hospital, Zibo, Shandong, People’s Republic of China.

